# Blood pressure and cardiac autonomic adaptations to isometric exercise training: A randomized sham‐controlled study

**DOI:** 10.14814/phy2.15112

**Published:** 2022-01-27

**Authors:** Anthony Decaux, Jamie J. Edwards, Harry T. Swift, Philip Hurst, Jordan Hopkins, Jonathan D. Wiles, Jamie M. O’Driscoll

**Affiliations:** ^1^ School of Psychology and Life Sciences Canterbury Christ Church University Kent UK

**Keywords:** blood pressure, isometric exercise

## Abstract

Isometric exercise training (IET) is increasingly cited for its role in reducing resting blood pressure (BP). Despite this, few studies have investigated a potential sham effect attributing to the success of IET, thus dictating the aim of the present study. Thirty physically inactive males (*n* = 15) and females (*n* = 15) were randomly assigned into three groups. The IET group completed a wall squat intervention at 95% peak heart rate (HR) using a prescribed knee joint angle. The sham group performed a parallel intervention, but at an intensity (<75% peak HR) previously identified to be inefficacious over a 4‐week training period. No‐intervention controls maintained their normal daily activities. Pre‐ and post‐measures were taken for resting and continuous blood pressure and cardiac autonomic modulation. Resting clinic and continuous beat‐to‐beat systolic (−15.2 ± 9.2 and −7.3 ± 5.6 mmHg), diastolic (−4.6 ± 5 and −4.5 ± 5.1), and mean (−7 ± 4.2 and −7.5 ± 5.3) BP, respectively, all significantly decreased in the IET group compared to sham and no‐intervention control. The IET group observed a significant decrease in low‐frequency normalized units of heart rate variability concurrent with a significant increase in high‐frequency normalized units of heart rate variability compared to both the sham and no‐intervention control groups. The findings of the present study reject a nonspecific effect and further support the role of IET as an effective antihypertensive intervention.

**Clinical Trials ID:** NCT05025202.

## Graphical abstract

Isometric exercise training is increasingly recognized as a powerful alternative exercise intervention for the management of hypertension. Our findings suggest that the clinically significant reductions in blood pressure following isometric exercise training are the result of the intervention and not due to other nonspecific factors, such as the placebo effect.

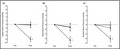

## 1 INTRODUCTION

Hypertension is well‐established as the leading modifiable risk factor for both cardiovascular disease and all‐cause mortality worldwide (Lim et al., 2012). The global prevalence of hypertension is estimated at 1.13 billion, which is associated with significant economic burden on healthcare services (Zhou et al., 2017). Isometric exercise training (IET) has emerged as a convenient, time‐efficient intervention, which has produced clinically significant blood pressure (BP) reductions in both hypertensive and normotensive populations (Inder et al., 2016). The antihypertensive effects of IET have been supported in multiple meta‐analytical studies (Carlson et al., 2014; Inder et al., 2016; López‐Valenciano et al., 2019), with reductions similar to or greater than those observed in traditional aerobic exercise training (Cornelissen & Smart, 2013).

While the efficacy of IET appears unequivocal, researchers have rarely evaluated this modality using rigorous research designs involving a placebo control, which is considered the gold standard for medical interventions (Fudim et al., 2019). The current evidence is therefore limited in determining whether the outcomes of IET are owing to the actual intervention or to other nonspecific factors, such as the placebo effect (Beedie et al., 2018; Hurst et al., 2019). The magnitude of the placebo effect on exercise interventions has been suggested to have a small to medium effect (Hurst et al., 2019) and can account for up to half of the observed psychological benefits of exercise (Lindheimer et al., 2015), as well as accounting for 34% and 47% of the antihypertensive drug response for systolic and diastolic BP, respectively (Wilhelm et al., 2016). Given the absence of appropriate placebo‐controlled studies, the efficacy of IET may be overestimated.

Controlling for nonspecific factors in exercise interventions is complicated by the inability to blind participants (i.e., participants are likely to be aware that they are, or they are not, receiving IET). Researchers have therefore advocated the use of sham controls resembling the intervention, but in a variant proven to be ineffective (Beedie et al., 2018; Lindheimer et al., 2015). To the best of our knowledge, there is only one IET study utilizing a sham design, in which the sham group performed a handgrip protocol, but was instructed not to generate any force during the exercise bouts (Ray & Carrasco, 2000). This design is problematic as the participants are likely to be aware that they are not performing the intervention and are therefore not sufficiently blinded. Thus, the application of a sham design IET intervention which effectively blinds the participants is imperative. It has previously been shown that 4 weeks of IET at 95% peak heart rate (peak HR) significantly improved resting blood pressure (Wiles et al., 2017), whereas 4 weeks of IET at 75% peak HR had no effect (Wiles et al., 2010). Given that these interventions are identical beside from the intensity, these results suggest that 4 weeks of IET at 75% peak HR, could be used as an appropriate sham for 4 weeks of IET at 95% peak HR.

In this study, we compared BP and cardiac autonomic modulation adaptations following 4 weeks of IET with 4 weeks of sham IET and a no‐intervention control. We hypothesized that the IET will reduce resting clinic and continuous beat‐to‐beat BP, along with improvements in cardiac autonomic modulation compared to sham and no‐intervention controls.

## 2 MATERIALS AND METHODS

### 2.1 Participants

Thirty physically inactive (self‐reported in accordance with the current guidelines) (World Health Organization, 2010) males (*n* = 15) and females (*n* = 15) were volunteered to participant in this study. Participants (age 30.2 ± 8.4 years; height 170.6 ± 9.2 cm; mass 82.3 ± 18.3 kg; BMI 28.2 ± 5.6 kg⋅m^−2^) were healthy with normal or high‐normal blood pressure under no pharmacotherapy, in accordance with the ESC/ESH guidelines for blood pressure classifications (<140/<90 mmHg) (Williams et al., 2018). All testing and data collection occurred at Canterbury Christ Church University. Informed consent was signed by all participants before testing. Canterbury Christ Church University Ethics Committee approved this research, ensuring conformity to the declaration of Helsinki principles (18/SAS/47C).

### 2.2 Resting clinic blood pressure

Participants were randomized into either the IET group, sham group, or no‐intervention control group through a single‐blinded protocol prior to any baseline measures. There were no significant differences in the participant physical characteristics between the groups (Table 1). Participants were required to refrain from strenuous exercise, caffeine, and alcohol consumption for 24 h and fast for 8 h prior to testing (Whelton et al., 2018). Participants attended the laboratory on two occasions for pre‐ and post‐intervention measures.

**TABLE 1:** Participant physical characteristics of the IET, control, and sham groups

Parameter	IET	Control	Sham
Age (years)	31.4 ± 6	28.3 ± 5.6	29.4 ± 7.8
Height (cm)	172 ± 11	170 ± 8.2	170 ± 8
Weight (kg)	83.7 ± 24	84.9 ± 21.7	79 ± 18
BMI (kg⋅m^−2^)	28.2 ± 7.8	29 ± 6.2	27.7 ± 5.8

Abbreviations: BMI, body mass index; IET, isometric exercise training.

Baseline resting systolic (sBP), mean (mBP), and diastolic (dBP) BP measures were recorded from the brachial artery as an average of three measures, separated by 5 min following 15 min of rest using an automated oscillometric BP monitor (Dinamap Pro 200 Critikon; GE Medical Systems, Freiburg, Germany) in accordance with the current guidelines (Whelton et al., 2018).

### 2.3 Continuous blood pressure and cardiac autonomics

Cardiac autonomic variables were measured using the Task Force^®^ Monitor (TFM), which is a validated noninvasive beat‐to‐beat monitoring system providing automatic calculations of all outputs. Using the TFM, continuous sBP, mBP, and dBP measures were acquired via the vascular unloading technique at the proximal limb of the index or middle finger, which was automatically corrected to oscillometric BP values obtained at the brachial artery of the opposite arm.

Heart rate (HR) was recorded through a six‐channel electrocardiogram and cardiac autonomic modulation was assessed by the oscillating fluctuations in the frequency and amplitude of each R‐R interval using power spectral analysis and applying an autoregressive model (Akselrod et al., 1981). Through the TFM’s automatic QRS algorithm, high‐ and low‐frequency parameters of heart rate variability were calculated and automatically expressed in both absolute (ms^2^) and normalized units (nu) (Li et al., 1995; Pan & Tompkins, 1985). All outcomes were acquired from a 5‐min recording period in the supine position as per recommended guidelines (Malik et al., 1996).

Baroreceptor reflex sensitivity was recorded via the sequence method which relies on the linear regression of continuous changes in sBP and the lengthening or shortening of the R‐R interval (Taylor et al., 2017). From all regressions, a mean slope of BRS was calculated and only sections with correlation coefficients of *r* > 0.95 were analyzed.

### 2.4 Isometric exercise training protocol

For the IET group, participants were required to complete a wall squat, consisting of resting their back against a fixed wall with their feet parallel, shoulder width aside, and their arms relaxed down by their side. As previously described (Goldring et al., 2014; O’Driscoll et al., 2017; Wiles et al., 2008), peak HR was determined via an incremental isometric wall squat test with beat‐to‐beat HR responses in accordance to the prescribed knee angle (Wiles et al., 2017). In line with previous evidence (Wiles et al., 2017), the intervention group was prescribed a 4‐week IET program at a knee joint angle predicted to elicit 95% peak HR. This intervention comprised of 4 × 2‐min bouts separated by 2‐min rest intervals, performed three times per week (12 IET sessions in total); ensuring a minimum of 48‐hour recovery between each session. To ensure that participants were working at the desired intensity, each participant was instructed to monitor HR throughout each session using a Polar RS400 (Polar Electro Oy, Professorintie 5, FIN‐90440 Kempele, Finland) HR monitor and report the HR data back to the researchers, in which the knee joint angle could be adjusted accordingly if required. Each participant used a “bend and squat” device (made in‐house), which was individually adjusted to govern the prescribed knee joint angle (Wiles et al., 2017).

For the sham group, participants performed the same incremental isometric wall squat test and parallel IET intervention. However, their training was prescribed at a knee joint angle, which would elicit an intensity of <75% peak HR so they did not achieve a sufficient physiological stimulus for BP adaptation to occur (Wiles et al., 2010). No‐intervention control participants were required to perform pre‐ and post‐measures, maintaining their normal routine and daily activities, which were confirmed prior to laboratory assessment.

### 2.5 Sample size

Based on previous studies utilizing wall squat isometric exercise training for BP reduction, we expected the IET intervention to result in a decrease in resting sBP of at least 6 mmHg (Taylor et al., 2019; Wiles et al., 2017) in the training group with no statistically significant change in the control group. This difference was considered to be clinically relevant. Using the likely changes and the coefficient of variation of sBP (4.6%) from Wiles et al. (2010), we estimated a sample size of 10 participants, with 80% power, and P less than 0.05.

### 2.6 Statistical analysis

Before analysis, all data were checked for conformity with parametric assumptions. All data were analyzed using SPSS (V22.0, release version for windows; Armonk, NY: IBM Corp) and presented as mean ± standard deviation. Comparison of data collected pre‐ and post‐intervention between the IET, sham, and no‐intervention control groups was analyzed using analysis of covariance (ANCOVA) with baseline parameters used as covariates to assess whether changes in BP and cardiac autonomic parameters following the intervention, sham, and no‐intervention control groups are influenced by initial baseline values. Statistical significance was deemed a priori as *p* < 0.05.

## 3 RESULTS

All thirty participants completed the study with no adverse events reported. Resting clinic HR, BP, continuous beat‐to‐beat BP, and cardiac autonomic variables were successfully acquired from all participants.

### 3.1 Resting clinic and continuous blood pressure

**FIGURE 1:**
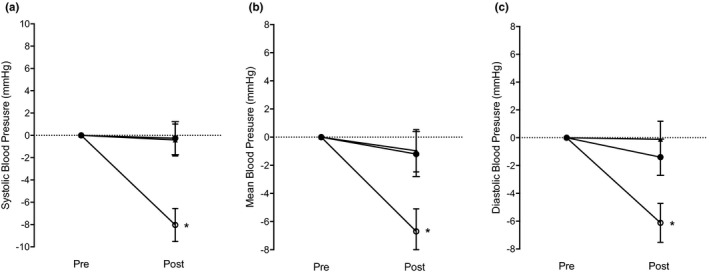
Mean continuous systolic (a), mean (b), and diastolic (c) blood pressure change values for the isometric exercise training group (open circles), no‐intervention control group (closed circles), and sham group (arrows). Note: Error bars indicate standard error of the mean; *p < 0.05 between the isometric exercise training group and both control and sham condition

**FIGURE 2:**
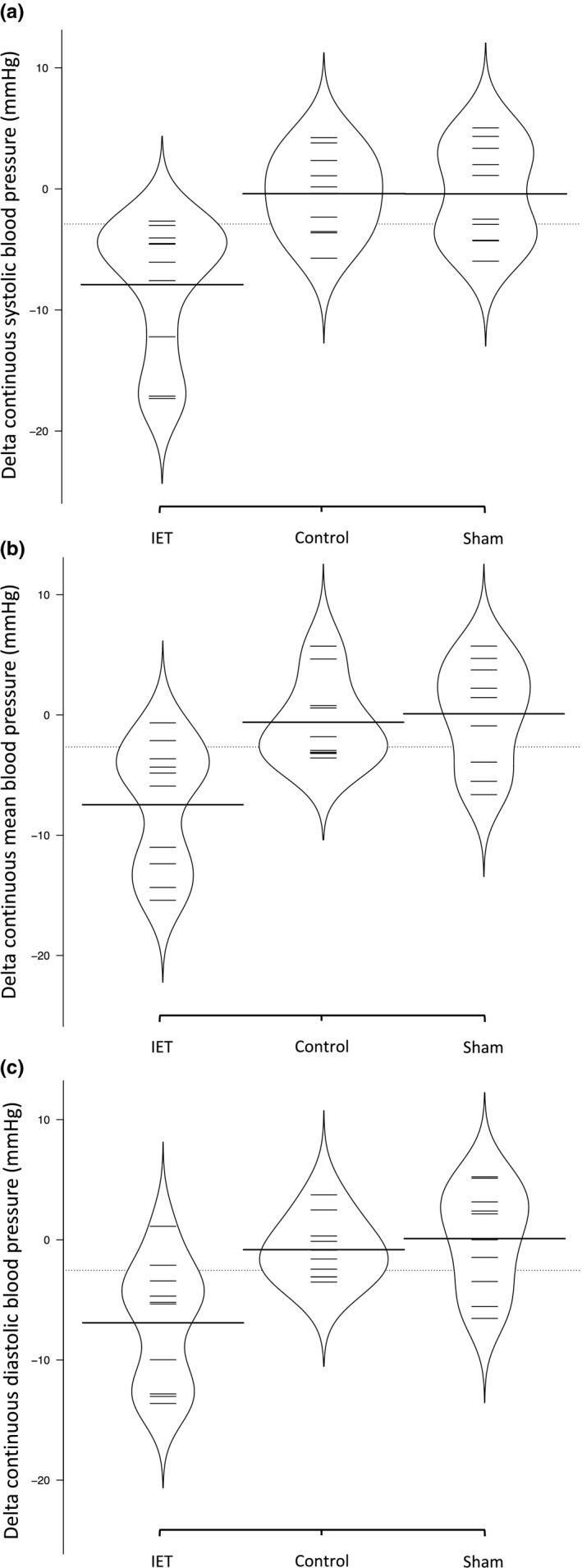
Illustrates the density distribution, average, and individual delta change in continuous systolic (a), mean (b), and diastolic (c) blood pressure following isometric exercise training, control, and sham groups

Participants in the IET group showed significant reductions in resting clinic sBP (−15 ± 9 mmHg, *p* = 0.003), mBP (−7 ± 4, *p* = 0.004), and dBP (−5 ± 5, *p* = 0.02) with no significant change in the sham (sBP −1 ± 5 mmHg, *p* = 0.98; mBP 0 ± 4, *p* = 0.72; and dBP 0 ± 2, *p* = 0.77) and no‐intervention control (sBP 1 ± 6 mmHg, *p* = 0.98; mBP 1 ± 4, *p* = 0.72; and dBP 1 ± 4, *p* = 0.77) groups (Table 2). Similarly, participants in the IET intervention showed significant reductions in continuous sBP (−7 ± 6 mmHg, *p* = 0.001), mBP (−8 ± 5 mmHg, *p* = 0.03), and dBP (−5 ± 5 mmHg, *p* = 0.004) with no significant changes in the sham (sBP 0 ± 4 mmHg, *p* = 0.94; mBP −1 ± 5, *p* = 0.91; and dBP 0 ± 4, *p* = 0.49) and no‐intervention control (sBP 0 ± 3 mmHg, *p* = 0.94; mBP −1 ± 3, *p* = 0.91; and dBP −1 ± 3, *p* = 0.49) groups (Table 2 and Figure 1). Figure 2 demonstrates the density distribution, mean, and individual changes in continuous sBP, mBP, and dBP following IET, control, and sham conditions.

**TABLE 2:** Resting blood pressure pre‐ and post‐isometric exercise training, control, and sham conditions

Parameter	IET (*n* = 10)		Control (*n* = 10)		Sham (*n* = 10)	
	Pre	Post	Pre	Post	Pre	Post
Clinic sBP (mmHg)	131 ± 6	116 ± 6*	119 ± 9	120 ± 7	120 ± 8	119 ± 8
Clinic mBP (mmHg)	97 ± 5	90 ± 5*	89 ± 5	90 ± 6	87 ± 2	89 ± 4
Clinic dBP (mmHg)	80 ± 6	75 ± 7*	73 ± 6	74 ± 8	71 ± 6	71 ± 6
Continuous sBP (mmHg)	117 ± 9	110 ± 13*	110 ± 9	110 ± 9	114 ± 4	114 ± 4
Continuous mBP (mmHg)	93 ± 8	85 ± 10*	84 ± 8	83 ± 8	87 ± 5	86 ± 4
Continuous dBP (mmHg)	65 ± 11	61 ± 11*	66 ± 9	66 ± 9	69 ± 6	69 ± 4

Abbreviation: dBP, diastolic blood pressure; IET, isometric exercise training; mBP, mean blood pressure; sBP, systolic blood pressure.

* *p *< 0.05.

### 3.2 Cardiac autonomic modulation

There was a significant decrease in low‐frequency normalized units (−12 ± 14%, *p* = 0.01) parallel to a significant increase in high‐frequency normalized units (12 ± 14%, *p* = 0.01) in the IET group compared to both the sham (5 ± 12%, *p* = 0.98 and −5 ± 12%, *p* = 0.98) and no‐intervention control (8 ± 7%, *p* = 0.98 and −8 ± 7%, *p* = 0.98) groups, for low frequency and high frequency, respectively. There were no differences in total power spectral density, absolute high frequency, absolute low‐frequency HRV, LF/HF ratio, HR, or BRS between IET, sham, and no‐intervention controls (Table 3).

**TABLE 3:** Cardiac autonomic parameters pre‐ and post‐isometric exercise training, control, and sham conditions

Parameter	IET (*n* = 10)		Control (*n* = 10)		Sham (*n* = 10)	
	Pre	Post	Pre	Post	Pre	Post
Heart rate (b⋅min^−1^)	68 ± 12	67 ± 10	70 ± 8	67 ± 10	78 ± 13	80 ± 13
PSD (ms^2^)	2332 ± 1804	2974 ± 2916	2604 ± 2824	2696 ± 2199	2591 ± 2319	2686 ± 2901
LF (ms^2^)	1109 ± 960	918 ± 637	883 ± 731	1029 ± 563	1000 ± 665	1195 ± 1053
HF (ms^2^)	933 ± 1057	1702 ± 2177	1227 ± 1528	1114 ± 1421	1029 ± 1120	1196 ± 1763
LF/HF ratio	1.52 ± 0.58	1.11 ± 0.62	1.22 ± 0.6	1.58 ± 0.75	1.41 ± 0.68	1.72 ± 1.02
LFnu (%)	60.1 ± 16	48.4 ± 18*	51.3 ± 13	59 ± 16	55.1 ± 13	60.5 ± 16
HFnu (%)	39.9 ± 16	51.6 ± 18*	48.7 ± 13	41 ± 16	44.9 ± 13	39.5 ± 16
BRS (ms⋅mmHg^−1^)	22.9 ± 12	26.3 ± 16	19.1 ± 7	19.2 ± 6	23.4 ± 11	21.4 ± 13

Abbreviations: BRS, baroreceptor reflex sensitivity; HF, high frequency; HFnu, normalized units high frequency; LF, low frequency; LF/HF ratio, low frequency to high frequency ratio; LFnu, normalized units low frequency; PSD, power spectral density.

* *p *< 0.05.

## 4 DISCUSSION

This study examined the efficacy of 4 weeks of IET on BP and cardiac autonomics in comparison to a sham and no‐intervention control. In line with our research hypothesis, we found that a 4‐week IET intervention significantly reduced resting clinic and continuous blood pressure measures compared to a 4‐week sham intervention and no‐intervention control group. These findings suggest that BP responses to IET are fundamentally intensity dependent, and that 75% peak HR is an intensity insufficient to elicit such responses over this training period duration.

In line with previous research (Paz et al., 2016), the observed reductions in both resting and continuous sBP, mBP, and dBP following the 4‐week IET intervention are clinically significant at a magnitude similar to that reported with antihypertensive pharmacotherapy (Law et al., 2009). Importantly, such results are associated with statistically significant reductions in risk of cardiovascular disease and mortality; providing further support for the clinical utility of IET in BP management (Brunström & Carlberg, 2018; Ettehad et al., 2016).

An important aspect of the current study was the inclusion of the sham control, which allowed us to delineate the specific and nonspecific effects of the intervention. Recent evidence has shown that many exercise and blood pressure interventions can be influenced through nonspecific effects, such as the placebo effect (Hurst et al., 2019; Lindheimer et al., 2015) and regression to the mean (Moore et al., 2019), which may overestimate the true effect of an intervention (Beedie et al., 2018). Participants in the sham control group performed the IET intervention at 75% peak HR for 4 weeks and reported no differences in any outcome variables when compared to participants in the no‐treatment control; while differences were observed for participants who completed 4 weeks of IET. These results support previous findings of the inefficacy of a 4‐week IET at 75% peak HR (Wiles et al., , 2017) and indicate its function as an appropriate sham control when used with this amount and duration of IET.

The significant BP reductions reported in the IET group compared to both sham and no‐intervention control groups suggest that the BP lowering effects of IET are directly attributable to physiological adaptations due to the specific physical training stimulus resulting from exceeding a threshold intensity of IE. Specifically, as supported in previous research (Goldring et al., 2014; O’Driscoll et al., 2017; Wiles et al., 2008), our data support adaptations in cardiac autonomic modulation as an important mechanistic pathway. Although debated (Goldstein et al., 2011), it is generally accepted that the low‐frequency component of HRV primarily represents sympathetic activity and high frequency predominantly represents parasympathetic outflow (Shaffer & Ginsberg, 2017). As such, the findings of this paper suggest an increase in cardiac vagal control with a decrease in sympathetic tone as a mechanistic pathway for the observed reduction in BP following IET (Prakash et al., 2005; Taylor et al., 2019). However, the changes in LF/HF ratio were not statistically significant and thus do not directly support this concept.

No significant differences in resting HR or BRS between IET, sham, and no‐intervention control suggest that other mechanisms are responsible for the observed reductions in BP. However, previous research has demonstrated that BRS may be a significant mechanistic pathway for the observed BP reductions (O’Driscoll et al., 2021; Taylor et al., 2017; Taylor et al., 2019). It is therefore likely that the present work was underpowered to detect significant changes in BRS.

Before exercise interventions can be adopted by society, it important that researchers use appropriate controls when evaluating their efficacy. However, a fundamental challenge in establishing efficacy is the development of appropriate sham controls that are indistinguishable from the true intervention and have no clinical benefit (Beedie et al., 2018; Hurst et al., 2019). In this study, we provide evidence that 4 weeks of IET at 75% peak HR can be used as a valid sham control for research investigating the efficacy of 4 weeks of IET at 95% peak HR. Research examining the efficacy of IET should adopt similar sham controls to improve accuracy of results. If these are not included, effects may be overestimated and owing to nonspecific factors, such as the placebo effect, which has been shown to significantly affect the outcome of exercise interventions (Hurst et al., 2019; Lindheimer et al., 2015). We therefore suggest that researchers investigating IET include sham controls in study design to make more accurate inferences about its efficacy.

### 4.1 Limitations and future research

It is important to consider the limitations of this study. First, the sample size is small and underpowered. However, it should be noted that this study is one of the first to show support for the efficacy of an IET sham intervention. A larger randomized sham‐controlled study should be performed in future, with measures of central (e.g., cardiac functional and mechanical responses) and peripheral (e.g., vascular function) parameters, to further ascertain a mechanistic adaptation for BP reduction. Second, baseline BP in the IET group was higher than both sham and no‐intervention control groups. Previous research has identified greater reductions in BP for those with higher baseline BP, thus potentially exaggerating our observed reductions in the IET group (Cornelissen & Smart, 2013; Hu et al., 2017). However, it was a randomized control study, and there were no significant baseline differences in continuous blood pressure measures between the groups. Thus, future research should aim to recruit a sample with more homogenous baseline characteristics. Furthermore, we sampled a healthy cohort with normal to high‐normal baseline BP and the relative application of our findings to diseased and hypertensive populations is unknown. While the safety of this IET protocol has previously been investigated in stage 1 hypertensives (Wiles et al., 2018), these findings do not extend to those with stage 2 hypertension and beyond. Researchers should consider replicating the results of our study on hypertensive participants. Finally, for a more rigorous sham design, future research should include a manipulation check and assess whether the participants in the sham group expected the intervention to be effective.

### 4.2 Conclusion

This randomized, between participant, sham‐controlled study supports the role of IET as an effective antihypertensive intervention. We found that BP and cardiac autonomic modulation improved following 4 weeks of IET at 95% peak HR than sham and no‐intervention control groups. These findings suggest that the effects of IET are the result of the intervention and are not to other nonspecific factors, such as the placebo effect. These results further support that IET produces clinically relevant reductions in both resting and continuous BP. Future research sampling a larger, hypertensive population is needed.

## CONFLICT OF INTEREST

There is no conflict of interest.

## AUTHOR CONTRIBUTIONS

The sharing of data in an open‐access repository was not included in our participants consent. Thus, in accordance with standard ethical practice, data may only be available upon request from the corresponding author.

## References

[phy215112-bib-0001] Akselrod, S. , Gordon, D. , Ubel, F. , Shannon, D. , Berger, A. , & Cohen, R. (1981). Power spectrum analysis of heart rate fluctuation: A quantitative probe of beat‐to‐beat cardiovascular control. Science, 213(4504), 220–222. 10.1126/science.6166045 6166045

[phy215112-bib-0002] Beedie, C. , Benedetti, F. , Barbiani, D. , Camerone, E. , Cohen, E. , Coleman, D. , Davis, A. , Elsworth‐Edelsten, C. , Flowers, E. , Foad, A. , Harvey, S. , Hettinga, F. , Hurst, P. , Lane, A. , Lindheimer, J. , Raglin, J. , Roelands, B. , Schiphof‐Godart, L. , & Szabo, A. (2018). Consensus statement on placebo effects in sports and exercise: The need for conceptual clarity, methodological rigour, and the elucidation of neurobiological mechanisms. European Journal of Sport Science, 18, 1383–1389. 10.1080/17461391.2018.1496144 30114971

[phy215112-bib-0004] Brunström, M. , & Carlberg, B. (2018). ‘Association of blood pressure lowering with mortality and cardiovascular disease across blood pressure levels a systematic review and meta‐analysis. JAMA Internal Medicine, 28–36. 10.1001/jamainternmed.2017.6015 29131895 PMC5833509

[phy215112-bib-0005] Carlson, D. J. , Dieberg, G. , Hess, N. C. , Millar, P. J. , & Smart, N. A. (2014). Isometric exercise training for blood pressure management: A systematic review and meta‐analysis. Mayo Clinic Proceedings, 89(3), 327–334. 10.1016/j.mayocp.2013.10.030 24582191

[phy215112-bib-0006] Cornelissen, V. A. , & Smart, N. A. (2013). Exercise training for blood pressure: A systematic review and meta‐analysis. Journal of the American Heart Association, 2, e004473. 10.1161/JAHA.112.004473 23525435 PMC3603230

[phy215112-bib-0007] Ettehad, D. , Emdin, C. A. , Kiran, A. , Anderson, S. G. , Callender, T. , Emberson, J. , Chalmers, J. , Rodgers, A. , & Rahimi, K. (2016). Blood pressure lowering for prevention of cardiovascular disease and death: A systematic review and meta‐analysis. The Lancet, 387(10022), 957–967. 10.1016/S0140-6736(15)01225-8 26724178

[phy215112-bib-0008] Fudim, M. , Ali‐Ahmed, F. , Patel, M. R. , & Sobotka, P. A. (2019). Sham trials: Benefits and risks for cardiovascular research and patients. The Lancet, 393(10186), 2104–2106. 10.1016/S0140-6736(19)31120-1 31226034

[phy215112-bib-0009] Goldring, N. , Wiles, J. D. , & Coleman, D. (2014). The effects of isometric wall squat exercise on heart rate and blood pressure in a normotensive population. Journal of Sports Sciences, 32(2), 129–136. 10.1080/02640414.2013.809471 23879248

[phy215112-bib-0010] Goldstein, D. S. , Bentho, O. , Park, M.‐Y. , & Sharabi, Y. (2011). Low‐frequency power of heart rate variability is not a measure of cardiac sympathetic tone but may be a measure of modulation of cardiac autonomic outflows by baroreflexes. Experimental Physiology, 96, 1255–1261. 10.1113/expphysiol.2010.056259 21890520 PMC3224799

[phy215112-bib-0012] Hu, H. , Zhang, J. , Wang, Y. , Tian, Z. , Liu, D. , Zhang, G. , Gu, G. , Zheng, H. , Xie, R. , & Cui, W. (2017). Impact of baseline blood pressure on the magnitude of blood pressure lowering by nifedipine gastrointestinal therapeutic system: Refreshing the wilder’s principle. Drug Design, Development and Therapy, 11, 3179–3186. 10.2147/DDDT.S143551 PMC5683797 29158664

[phy215112-bib-0013] Hurst, P. , Schipof‐Godart, L. , Szabo, A. , Raglin, J. , Hettinga, F. , Roelands, B. , Lane, A. , Foad, A. , Coleman, D. , & Beedie, C. (2019). The Placebo and Nocebo effect on sports performance: A systematic review. European Journal of Sport Science, 20(3), 279–292. 10.1080/17461391.2019.1655098 31414966

[phy215112-bib-0014] Inder, J. D. , Carlson, D. J. , Dieberg, G. , McFarlane, J. R. , Hess, N. C. L. , & Smart, N. A. (2016). Isometric exercise training for blood pressure management: A systematic review and meta‐analysis to optimize benefit. Hypertension Research, 39(2), 89–94. 10.1038/hr.2015.111 26467494

[phy215112-bib-0016] Law, M. R. , Morris, J. K. , & Wald, N. J. (2009). Use of blood pressure lowering drugs in the prevention of cardiovascular disease: Meta‐analysis of 147 randomised trials in the context of expectations from prospective epidemiological studies. BMJ, 338(7705), 1245. 10.1136/bmj.b1665 PMC2684577 19454737

[phy215112-bib-0017] Li, C. , Zheng, C. , & Tai, C. (1995). Detection of ECG characteristic points using wavelet transforms. IEEE Transactions on Biomedical Engineering, 42(1), 21–28. 10.1109/10.362922 7851927

[phy215112-bib-0018] Lim, S. S. , Vos, T. , Flaxman, A. D. , Danaei, G. , Shibuya, K. , Adair‐Rohani, H. , AlMazroa, M. A. , Amann, M. , Anderson, H. R. , Andrews, K. G. , Aryee, M. , Atkinson, C. , Bacchus, L. J. , Bahalim, A. N. , Balakrishnan, K. , Balmes, J. , Barker‐Collo, S. , Baxter, A. , Bell, M. L. , … Ezzati, M. (2012). A comparative risk assessment of burden of disease and injury attributable to 67 risk factors and risk factor clusters in 21 regions, 1990–2010: A systematic analysis for the Global Burden of Disease Study 2010. The Lancet, 380(9859), 2224–2260. 10.1016/S0140-6736(12)61766-8 PMC4156511 23245609

[phy215112-bib-0019] Lindheimer, J. B. , O’Connor, P. J. , & Dishman, R. K. (2015). Quantifying the placebo effect in psychological outcomes of exercise training: A meta‐analysis of randomized trials. Sports Medicine, 45, 693–711. 10.1007/s40279-015-0303-1 25762083

[phy215112-bib-0020] López‐Valenciano, A. , Ruiz‐Pérez, I. , Ayala, F. , Sánchez‐Meca, J. , & Vera‐Garcia, F. J. (2019). Updated systematic review and meta‐analysis on the role of isometric resistance training for resting blood pressure management in adults. Journal of Hypertension, 37, 1320–1333. 10.1097/HJH.0000000000002022 30624369

[phy215112-bib-0021] Malik, M. , Thomas Bigger, J. , John Camm, A. , Kleiger, R. E. , Malliani, A. , Moss, A. J. , & Schwartz, P. J. (1996). Heart rate variability: Standards of measurement, physiological interpretation, and clinical use. Circulation, 93(5), 1043–1065. 10.1161/01.cir.93.5.1043 8598068

[phy215112-bib-0022] Moore, M. N. , Atkins, E. R. , Salam, A. , Callisaya, M. L. , Hare, J. L. , Marwick, T. H. , Nelson, M. R. , Wright, L. , Sharman, J. E. , & Rodgers, A. (2019). Regression to the mean of repeated ambulatory blood pressure monitoring in five studies. Journal of Hypertension, 37(1), 24–29. 10.1097/HJH.0000000000001977 30499921

[phy215112-bib-0041] O'Driscoll, J. M. , Boucher, C. , Vilda, M. , Taylor, K. A. , & Wiles, J. D. (2021). Continuous cardiac autonomic and haemodynamic responses to isometric exercise in females. European Journal of Applied Physiology, 121(1), 319–329. 10.1007/s00421-020-04525-z 33070245

[phy215112-bib-0024] O'Driscoll, J. M. , Taylor, K. A. , Wiles, J. D. , Coleman, D. A. , & Sharma, R. (2017). Acute cardiac functional and mechanical responses to isometric exercise in prehypertensive males. Physiological Reports, 5(7), 10.14814/phy2.13236 PMC5392522 28381447

[phy215112-bib-0025] Pan, J. , & Tompkins, W. J. (1985). A real‐time QRS detection algorithm. IEEE Transactions on Biomedical Engineering, BME‐32, 230–236. 10.1109/TBME.1985.325532 3997178

[phy215112-bib-0026] Paz, M. A. , de‐La‐Sierra, A. , Sáez, M. , Barceló, M. A. , Rodríguez, J. J. , Castro, S. , Lagarón, C. , Garrido, J. M. , Vera, P. , & Coll‐de‐Tuero, G. (2016). Treatment efficacy of anti‐hypertensive drugs in monotherapy or combination: ATOM systematic review and meta‐analysis of randomized clinical trials according to PRISMA statement. Medicine, 95(30), e4071. 10.1097/MD.0000000000004071 27472680 PMC5265817

[phy215112-bib-0027] Prakash, E. S. , Sethuraman, K. R. , & Narayan, S. K. (2005). Cardiovascular autonomic regulation in subjects with normal blood pressure, high‐normal blood pressure and recent‐onset hypertension. Clinical and Experimental Pharmacology and Physiology, 32, 488–494. 10.1111/j.1440-1681.2005.04218.x 15854164

[phy215112-bib-0028] Ray, C. A. , & Carrasco, D. I. (2000). Isometric handgrip training reduces arterial pressure at rest without changes in sympathetic nerve activity. Available at: http://www.ajpheart.org (Accessed: 23 April 2021). 10.1152/ajpheart.2000.279.1.H245 10899063

[phy215112-bib-0029] Shaffer, F. , & Ginsberg, J. P. (2017). An overview of heart rate variability metrics and norms. Frontiers in Public Health, 5, 258. 10.3389/fpubh.2017.00258 29034226 PMC5624990

[phy215112-bib-0030] Taylor, K. A. , Wiles, J. D. , Coleman, D. A. , Leeson, P. , Sharma, R. , & O’Driscoll, J. M. (2019). Neurohumoral and ambulatory haemodynamic adaptations following isometric exercise training in unmedicated hypertensive patients. Journal of Hypertension, 37(4), 827–836. 10.1097/HJH.0000000000001922 30817465

[phy215112-bib-0031] Taylor, K. A. , Wiles, J. D. , Coleman, D. D. , Sharma, R. , & O'driscoll, J. M. (2017). Continuous cardiac autonomic and hemodynamic responses to isometric exercise. Medicine and Science in Sports and Exercise, 49(8), 1511–1519. 10.1249/MSS.0000000000001271 28708775

[phy215112-bib-0032] Whelton, P. K. , Carey, R. M. , Aronow, W. S. , Casey, D. E. Jr , Collins, K. J. , Dennison Himmelfarb, C. , DePalma, S. M. , Gidding, S. , Jamerson, K. A. , Jones, D. W. , MacLaughlin, E. J. , Muntner, P. , Ovbiagele, B. , Smith, S. C. Jr , Spencer, C. C. , Stafford, R. S. , Taler, S. J. , Thomas R. J. , Williams, K. A. Sr , … Wright, J. T. Jr (2018). 2017 ACC/AHA/AAPA/ABC/ACPM/AGS/APhA/ASH/ASPC/NMA/PCNA guideline for the prevention, detection, evaluation, and management of high blood pressure in adults: Executive summary. Journal of the American Society of Hypertension, 12, (8), 579.e1. –579.e73. 10.1016/j.jash.2018.06.010 30219548

[phy215112-bib-0033] Wiles, J. D. , Allum, S. R. , Coleman, D. A. , & Swaine, I. L. (2008). The relationships between exercise intensity, heart rate, and blood pressure during an incremental isometric exercise test. Journal of Sports Sciences, 26(2), 155–162. 10.1080/02640410701370655 17852666

[phy215112-bib-0034] Wiles, J. D. , Coleman, D. A. , & Swaine, I. L. (2010). The effects of performing isometric training at two exercise intensities in healthy young males. European Journal of Applied Physiology, 108(3), 419–428. 10.1007/s00421-009-1025-6 19280213

[phy215112-bib-0036] Wiles, J. D. , Goldring, N. , & Coleman, D. (2017). Home‐based isometric exercise training induced reductions resting blood pressure. European Journal of Applied Physiology, 117(1), 83–93. 10.1007/s00421-016-3501-0 27853886

[phy215112-bib-0037] Wiles, J. D. , Taylor, K. , Coleman, D. , Sharma, R. , & O'Driscoll, J. (2018). The safety of isometric exercise: Rethinking the exercise prescription paradigm for those with stage 1 hypertension. Medicine, 97(10), e0105. 10.1097/MD.0000000000010105 29517686 PMC5882444

[phy215112-bib-0038] Wilhelm, M. , Winkler, A. , Rief, W. , & Doering, B. K. (2016). Effect of placebo groups on blood pressure in hypertension: A meta‐analysis of beta‐blocker trials. Journal of the American Society of Hypertension, 10(12), 917–929. 10.1016/j.jash.2016.10.009 27865824

[phy215112-bib-0042] Williams, B. , Mancia, G. , Spiering, W. , Rosei, E. A. , Azizi, M. , Burnier, M. , Clement, D. L. , Coca, A. , de Simone, G. , Dominiczak, A. , Kahan, T. , Mahfoud, F. , Redon, J. , Ruilope, L. , Zanchetti, A. , Kerins, M. , Kjeldsen, S. E. , Kreutz, R. , Laurent, S. , … ESC Scientific Document Group . (2018). ESC/ESH guidelines for the management of arterial hypertension: The task force for the management of arterial hypertension of the European Society of Cardiology (ESC) and the European Society of Hypertension (ESH). European Heart Journal, 39(33), 3021–3104. 10.1093/eurheartj/ehy339 30165516

[phy215112-bib-0039] World Health Organization . (2010) Global recommendations on physical activity for health. World Health Organization. World Health Organization. Available at: http://scholar.google.com/scholar?hl=en&btnG=Search&q=intitle:Global+Recommendations+on+Physical+Activity+for+Health#0 (Accessed: 3 April 2020).

[phy215112-bib-0040] Zhou, B. , Bentham, J. , Di Cesare, M. , Bixby, H. , Danaei, G. , Cowan, M. J. , Paciorek, C. J. , Singh, G. , Hajifathalian, K. , Bennett, J. E. , Taddei, C. , Bilano, V. , Carrillo‐Larco, R. M. , Djalalinia, S. , Khatibzadeh, S. , Lugero, C. , Peykari, N. , Zhang, W. Z. , Lu, Y. , … Zuñiga Cisneros, J. (2017). Worldwide trends in blood pressure from 1975 to 2015: a pooled analysis of 1479 population‐based measurement studies with 19·1 million participants. The Lancet, 389(10064), 37–55. 10.1016/S0140-6736(16)31919-5 PMC5220163 27863813

